# Comparative analysis of convolutional neural networks and traditional machine learning models for IVF live birth prediction: a retrospective analysis of 48514 IVF cycles and an evaluation of deployment feasibility in resource-constrained settings

**DOI:** 10.3389/fendo.2025.1556681

**Published:** 2025-06-12

**Authors:** Yu Liu, Yi Wang, Kai Huang, Hao Shi, Hang Xin, Shanjun Dai, Jinhao Liu, Xinhong Yang, Jianyuan Song, Fuli Zhang, Yihong Guo

**Affiliations:** Reproductive Medicine Center, The First Affiliated Hospital of Zhengzhou University, Zhengzhou, China

**Keywords:** assisted reproductive technology, machine learning, deep learning, convolutional neural network, artificial intelligence, resource-limited settings, model interpretability

## Abstract

**Objective:**

To evaluate the predictive performance of a convolutional neural network for analyzing electronic medical records in assisted reproductive therapy and to compare its accuracy and interpretability with traditional machine learning models. The study also explores the feasibility of deploying such models in resource-limited clinical settings.

**Design:**

Retrospective cohort study based on EMR data using five models: CNN, Naïve Bayes, Random Forest, Decision Tree, and Feedforward Neural Network. Feature importance and model interpretability were evaluated using SHAP.

**Setting:**

First Hospital of Zhengzhou University.

**Population:**

48,514 fresh IVF cycles from August 2009 to May 2018.

**Methods:**

Preprocessed EMR data were used to train and evaluate five classification models predicting live birth outcomes. Stratified 5-fold cross-validation was performed for robust performance estimation. ROC curves and AUC values were used for comparative evaluation.

**Main Outcome Measure:**

Live birth.

**Results:**

The CNN model achieved an accuracy of 0.9394 ± 0.0013, AUC of 0.8899 ± 0.0032, precision of 0.9348 ± 0.0018, recall of 0.9993 ± 0.0012, and F1 score of 0.9660 ± 0.0007. Its performance was comparable to Random Forest (accuracy: 0.9406 ± 0.0017, AUC: 0.9734 ± 0.0012), and superior to Decision Tree, Naïve Bayes, and Feedforward Neural Network in recall and robustness. CNN demonstrated stable convergence during training, and SHAP-based interpretation highlighted maternal age, BMI, antral follicle count, and gonadotropin dosage as the top predictors for live birth outcome.

**Conclusions:**

With appropriate input transformation, CNNs can effectively model structured EMR data and offer predictive performance comparable to ensemble methods. Their scalability, high sensitivity, and interpretability make CNNs promising candidates for integration into clinical workflows, particularly in environments with limited computational resources.

## Introduction

*In vitro* fertilization (IVF), a cornerstone of assisted reproductive technology (ART), has brought hope to millions of couples experiencing infertility. Despite its transformative impact, the overall live birth rate per cycle remains suboptimal—often below 40% globally—largely influenced by patient-specific factors such as maternal age, infertility duration, and ovarian reserve, as reported in large-scale epidemiological studies (1, 2). Accurate prediction of IVF success is essential for optimizing clinical decision-making, improving resource allocation, and managing patient expectations (3).

Electronic medical records (EMRs), which store detailed patient information including demographic, hormonal, and procedural data, offer an unparalleled opportunity to build predictive models for IVF outcomes (4). Over the past decade, machine learning models have demonstrated potential in identifying patterns within EMRs to enhance IVF prediction (5, 6). Traditional methods, such as logistic regression and decision trees, have been widely applied due to their interpretability and computational efficiency (7). However, these models often struggle with high-dimensional data and fail to capture complex, nonlinear interactions (8).

Recent advancements in deep learning, particularly convolutional neural networks (CNNs), have enabled the automatic extraction of intricate patterns from structured and unstructured data (9, 10). CNNs excel in image-based tasks but are increasingly applied to tabular EMR data, offering improved predictive power compared to traditional models (11). Despite these advantages, challenges remain, including the high computational requirements of CNNs and their dependence on large datasets, which may limit their application in resource-constrained environments (12, 13). While the feasibility of CNNs in IVF prediction has been explored, few studies have systematically compared their performance with traditional machine learning models (6). Furthermore, the deployment of predictive models in resource-limited settings, where computational and human resources are often constrained, has received little attention. Addressing these gaps is critical for the development of scalable, clinically relevant solutions (9, 14).

This study aims to bridge these gaps by conducting a large-scale retrospective analysis of EMR data from 48514 IVF cycles. Specifically, we compare the performance of CNNs and traditional machine learning models in predicting live birth outcomes. Additionally, we assess the feasibility of deploying these models in resource-limited settings, offering insights into their real-world applicability in reproductive medicine.

## Patient selection

This study included patients who underwent fresh IVF cycles at the First Affiliated Hospital of Zhengzhou University between August 2009 and May 2018. A total of 48514 patients were enrolled in the cohort.

## Sample size estimation

In this study, we estimated the required sample size using the formula n=Zα22*P*(1−P)d2 (15), where zα22 represents the critical value for a 95% confidence interval (1.96), *p* is the estimated prevalence of infertility in the population, and d denotes the margin of error. Based on the recent data published in jama in 2023 (16), we used 17% as the population incidence of infertility. A margin of error of 5% was selected to balance precision and sample size feasibility. To account for potential loss to follow-up, we adjusted the sample size using $n_{adjusted}=\frac{n}{1-loss\;to\;follow-up\;rate}$ (15), assuming a loss to follow-up rate of 5%. The required sample size is about 228. Our final sample size of 48514 patients far exceeds the required minimum of 228, ensuring robust statistical power for this study. This approach ensures the robustness of our study’s statistical power.

## Data preprocessing and model implementation

All fresh IVF cycle data were extracted from the EMR system and underwent a standardized preprocessing workflow. Continuous variables with missing values were imputed using the mean, while categorical variables with missing entries were excluded only if they exceeded 50% missingness across the entire dataset. This threshold was set to reduce imputation bias and ensure model stability, based on established practices in clinical machine learning.

Categorical variables were transformed using one-hot encoding, applied prior to normalization. All numerical features were normalized to the range [-1, 1] using min-max scaling to standardize the feature space and ensure comparable weight contribution across models.

The final dataset was randomly divided into training (80%) and testing (20%) subsets, stratified by the outcome variable (live birth) to preserve class distribution. In addition, 5-fold cross-validation was employed on the training set to tune hyperparameters and validate model performance, ensuring generalizability and mitigating sampling bias.

## CNN input format and architecture

To adapt CNNs for structured clinical data, we first organized EMRs into two-dimensional matrices, where each row represented a patient and each column corresponded to a specific clinical feature. These matrices were then reshaped into single-channel pseudo-images with a fixed input shape of (1, 6, 7)—corresponding to 42 selected features arranged in a 7×6 grid—to enable convolutional kernels to capture local feature patterns and inter-feature dependencies.

A customized CNN was constructed comprising two convolutional layers with 16 and 32 filters (kernel size: 3×3), each followed by a ReLU activation and 2×2 max pooling to downsample feature maps. A dropout layer (rate = 0.5) was incorporated after the convolutional blocks to mitigate overfitting. The output feature maps were flattened and passed through two fully connected layers (64 and 1 units), with sigmoid activation applied at the output layer to produce live birth probability predictions.

To dynamically accommodate the input dimensionality, a dummy input tensor of shape (1, 1, 6, 7) was used during initialization to automatically determine the flattening dimension prior to the fully connected layers. Model training was conducted using PyTorch (v2.5), with binary cross-entropy loss, the Adam optimizer (learning rate: 0.001), and a batch size of 64. Early stopping was employed based on validation loss to prevent overfitting and enhance generalizability.

## Data collection and selection

**Figure 1:**
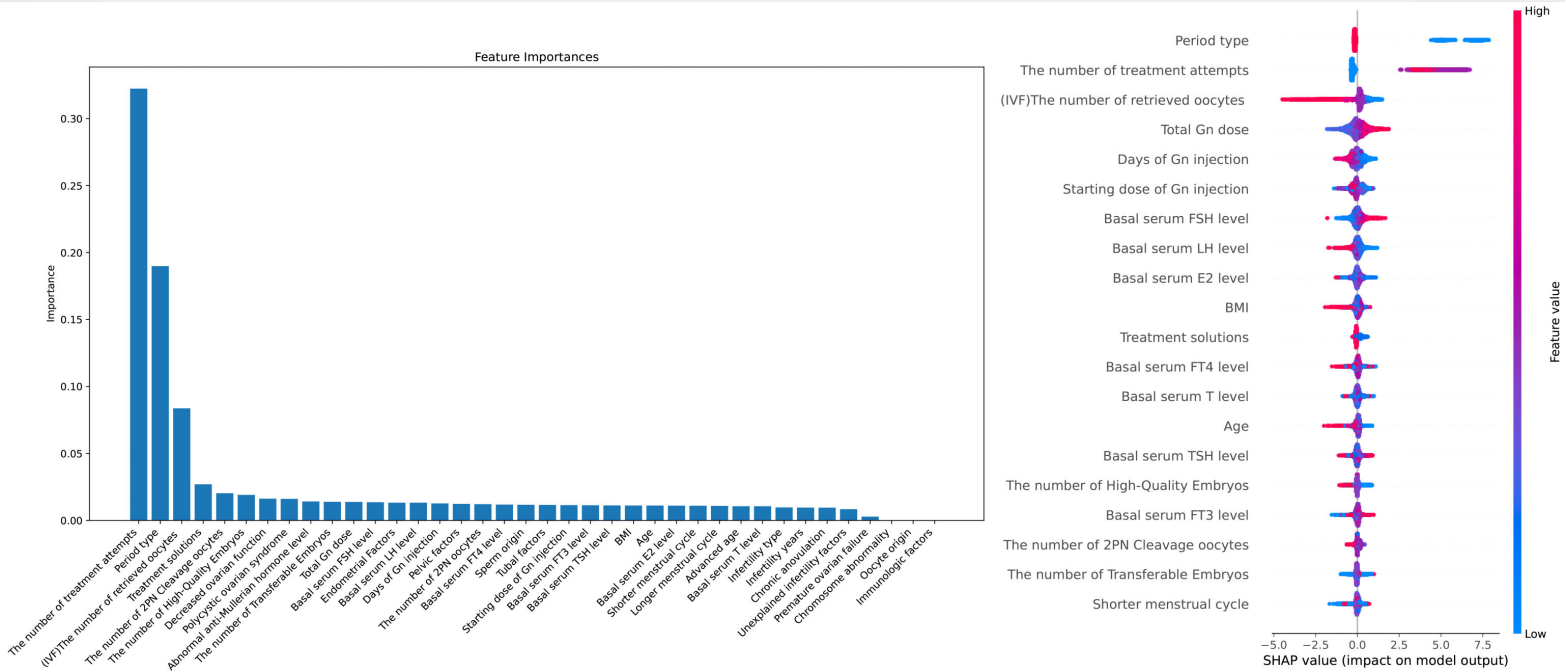
Feature Importance and Interpretability in Predicting IVF Outcomes: A Combined XGBoost and SHAP Analysis Notes: X-axis (SHAP value): Represents the impact of each feature on the model's prediction. Positive values indicate a feature pushes the prediction towards a positive outcome, while negative values indicate a negative influence. Y-axis (Feature names): Lists the model's input features, with higher-ranked features having a greater overall impact on the prediction. Color (Feature value): Indicates the actual value of the feature, where blue represents lower values and red represents higher values. The color helps to interpret the effect of different feature values on the model's output.

Data collection and entry into the electronic medical record system is done by professionally trained nurses in our center. XGBoost algorithm exhibits significant advantages in feature weight analysis. Its built-in feature importance evaluation method is capable of considering complex interactions among features (5) and improving the model’s robustness and generalization ability through ensemble learning (6). Furthermore, XGBoost’s feature importance scores can be utilized for feature selection and dimensionality reduction (7), enhancing the model’s interpretability and efficiency. Additionally, XGBoost provides intuitive visualization techniques that aid in understanding the model’s decision-making process (8). As shown in **Figure 1**, to enhance the interpretability of clinical feature selection and the robustness of the machine learning model, we used the XGBoost algorithm to rank the importance of clinical features in predicting the outcomes. The following are the clinical indicators we have selected for predicting live birth outcomes: “Female’s age”, “Types of infertility”, “Duration of infertility(years)”, “Shortest menstrual cycle(days)”, “Longest menstrual cycle(days)”, “Body Mass Index (BMI)”, “Basal blood Follicle-Stimulating Hormone (FSH) level”, “Basal blood Estradiol (E2) level”, “Basal blood Luteinizing Hormone (LH) level”, “Basal blood testosterone(T) level”, “Basal blood Free Triiodothyronine (FT3) level”, “Basal blood Free Thyroxine (FT4) level”, “Basal blood Thyroid-Stimulating Hormone (TSH) level”, “Unexplained infertility”, “Polycystic ovarian syndrome”, “Advanced age”, “Decreased ovarian function”, “Premature ovarian failure”, “Chronic anovulation”, “Pelvic factor (including chronic pelvic inflammatory disease and pelvic factors)”, “Immunologic factors”, “Abnormal anti-Mullerian hormone level”, “Tubal factor”, “Endometrial Factor”, “Chromosome abnormality”, “Sperm origin”, “Oocyte origin”, “Period type”, “Number of treatment attempts”, “Treatment solutions”, “Starting dose of Gn injection”, “Total dose of Gn injection”, “Days of Gn injection”, “Number of retrieved oocytes”, “Number of 2PN oocytes”, “Number of 2PN cleavage oocytes”, “Number of transferable embryos”, “Number of high-quality embryos”.

## Model interpretability

To improve the interpretability of our machine learning models, we employed SHAP, which provides insights into feature contributions to the predictions (8). This method has proven highly effective in enhancing the transparency of machine learning models in clinical settings, making the models more interpretable for healthcare professionals (9).

## Software and hardware

The programming language used for this experiment is PyTorch (https://pytorch.org/) and All analyses were conducted using Python 3.8 on a machine with an Intel^®^ Core™ i7-13700K Processor, and the graphics card was an NVIDIA^®^ GeForce RTX™ 3090 fitted with GPU. Key libraries included PyTorch (version 2.5, https://pytorch.org/), scikit-learn (version 1.6.0), and SHAP (version 0.39.0). We also tested model inference on systems with Apple M1/M2 chips, and found the trained CNN models could be deployed locally without GPU acceleration, requiring only 80–100 MB of memory and <0.05s per prediction. These results demonstrate that CNNs trained on structured EMR data are feasible for real-world deployment, even in computationally constrained environments.

## Controlled hyper-stimulation induction

All patients received one of the following four controlled ovarian stimulation (COS) regimens, which have been described previously (17): GnRH Antagonist Protocol, GnRH Agonist Protocol, Mild Stimulation Protocol, Ultra-long Protocol. The clinician selected the appropriate protocol for each patient on an individual basis according to the patient characteristics (17).

## Assessment methods

To systematically evaluate and compare the performance of the predictive models, we employed five-fold cross-validation on the training dataset. Evaluation metrics included accuracy, precision, recall, F1-score, and the area under the receiver operating characteristic curve (AUC). For each model, the mean and standard deviation of these metrics across five folds were reported to ensure robust statistical comparison (14). The evaluation metrics were defined as follows:


$$Accuracy=\frac{TP+TN}{TP+TN+FP+FN}$$

$$Precision=\frac{TP}{TP+FP}$$

$$Recall=\frac{TP}{TP+FN}$$

$$F1-score=\frac{2\times Precision\times Recall}{Precision+Recall}$$

Here, *TP* (true positive) represents the number of positive cases correctly classified as positive. *TN, FN*, and *FP* represent the number of true negative, false negative, and false positive cases, respectively. *Recall* represents the percentage of positive samples correctly classified, and *F1-score* is the weighted average of *precision* and *recall*, representing overall performance. The confusion matrix is a specific contingency table that allows for visualization of clinical relevance. Each point on the ROC curve reflects sensitivity to the same signal stimulus (12).

## Results

**Figure 2:**
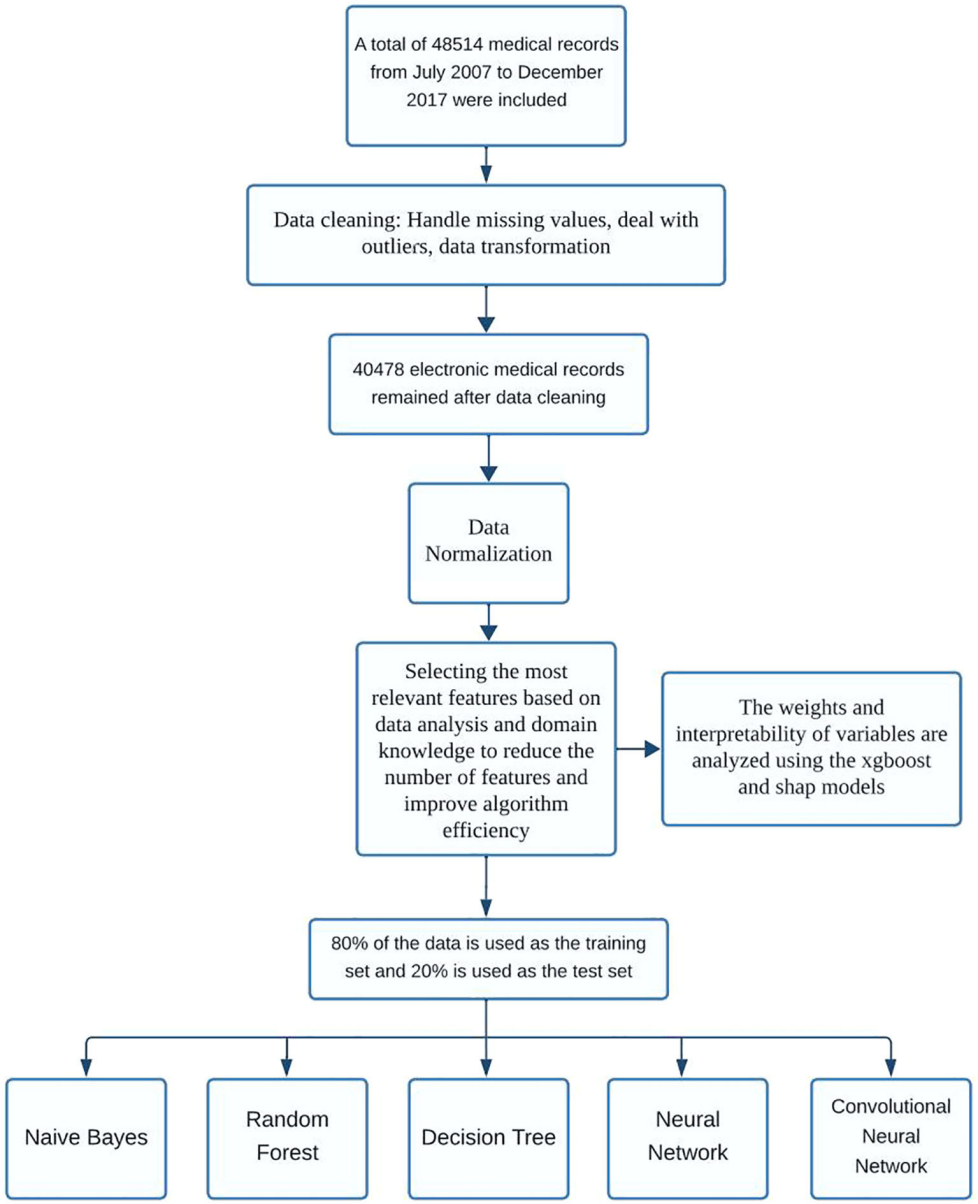
Flowchart of the study.

As illustrated in **Figure 2**, the overall study workflow included four major phases: data preprocessing, model construction, performance evaluation, and comparative analysis.

Initially, a retrospective dataset was assembled from the EMRs of patients undergoing *in vitro* fertilization (IVF). Although a more recent dataset containing over 50,000 records was initially considered, subsequent inspection revealed a high rate of missing values across key clinical variables, limiting its suitability for robust machine learning modeling. As a result, we retained the previously curated dataset containing 48,514 IVF cycles, which included 39 high-quality clinical and laboratory features with manageable missingness.

In the preprocessing stage, features with excessive missingness (>50%) were removed. Remaining continuous variables were standardized, while categorical variables were encoded using one-hot transformation. Binary outcome labels (live birth vs. non-live birth) were used for supervised classification.

Subsequently, five predictive models were constructed: Random Forest, Decision Tree, Naive Bayes, Feedforward Neural Network, and Convolutional Neural Network (CNN). To facilitate CNN modeling on tabular EMR data, an input reshaping strategy was applied to transform the structured features into a two-dimensional matrix, thereby enabling convolutional layer processing.

For evaluation, models were trained on 80% of the dataset and assessed using 5-fold cross-validation. Key metrics included area under the receiver operating characteristic curve (AUC), accuracy, precision, recall, and F1 score. ROC curves were plotted using the held-out test set to support visual comparison of discrimination ability (18).

Finally, comparative performance across models was interpreted with a focus on balancing classification accuracy, generalizability, and scalability. Particular attention was given to the CNN model, whose performance under class imbalance and structural adaptation was critically assessed.

In **Figure 1**, the left XGBoost plot illustrates the importance of various features in influencing outcomes, while the right SHAP plot assesses each feature’s contribution by averaging its impact when combined with others. For example, an increase in “The number of treatment attempts” is associated with a higher live birth rate per cycle, likely due to a higher probability of success with more attempts, as clinicians continuously refine personalized treatment plans based on the patient’s condition. Regarding “The number of retrieved oocytes,” a higher retrieval count in fresh cycles is associated with a lower live birth rate, likely due to the increased risk of ovarian hyperstimulation syndrome (OHSS) and subsequent cycle cancellations. Future models could consider cumulative live birth outcomes from single retrieval cycles for deeper insights. “Total Gn dose” is positively correlated with live birth rates, which may reflect better ovarian response and a higher number of retrieved oocytes. For “Basal serum FSH level” and “Basal serum LH level,” slightly elevated FSH levels and lower LH levels, where FSH is slightly higher than LH, appear to favor a higher live birth rate. Additionally, a “Shorter menstrual cycle” is associated with lower live birth rates, potentially due to the suboptimal endometrial environment linked to shorter cycles. Finally, both “BMI” and “Age” show similar patterns, with a significant decrease in live birth rates per cycle as these values increase.

**Figure 3:**
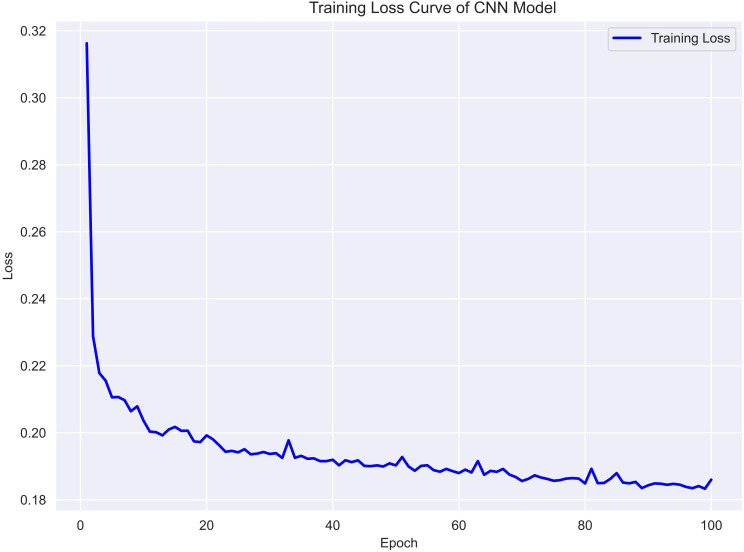
Training loss curve of CNN Model.

To assess the training dynamics of the CNN model, the binary cross-entropy loss was monitored throughout the training process. As shown in **Figure 3**, the training loss decreased steadily over 100 epochs, indicating effective convergence without signs of overfitting. The consistent downward trend suggests that the model was able to progressively capture discriminative patterns within the reshaped EMR input. No sudden spikes or fluctuations were observed, further supporting the stability of the optimization process. This learning curve supports the CNN model’s ability to generalize from structured clinical data with moderate complexity.

**Figure 4:**
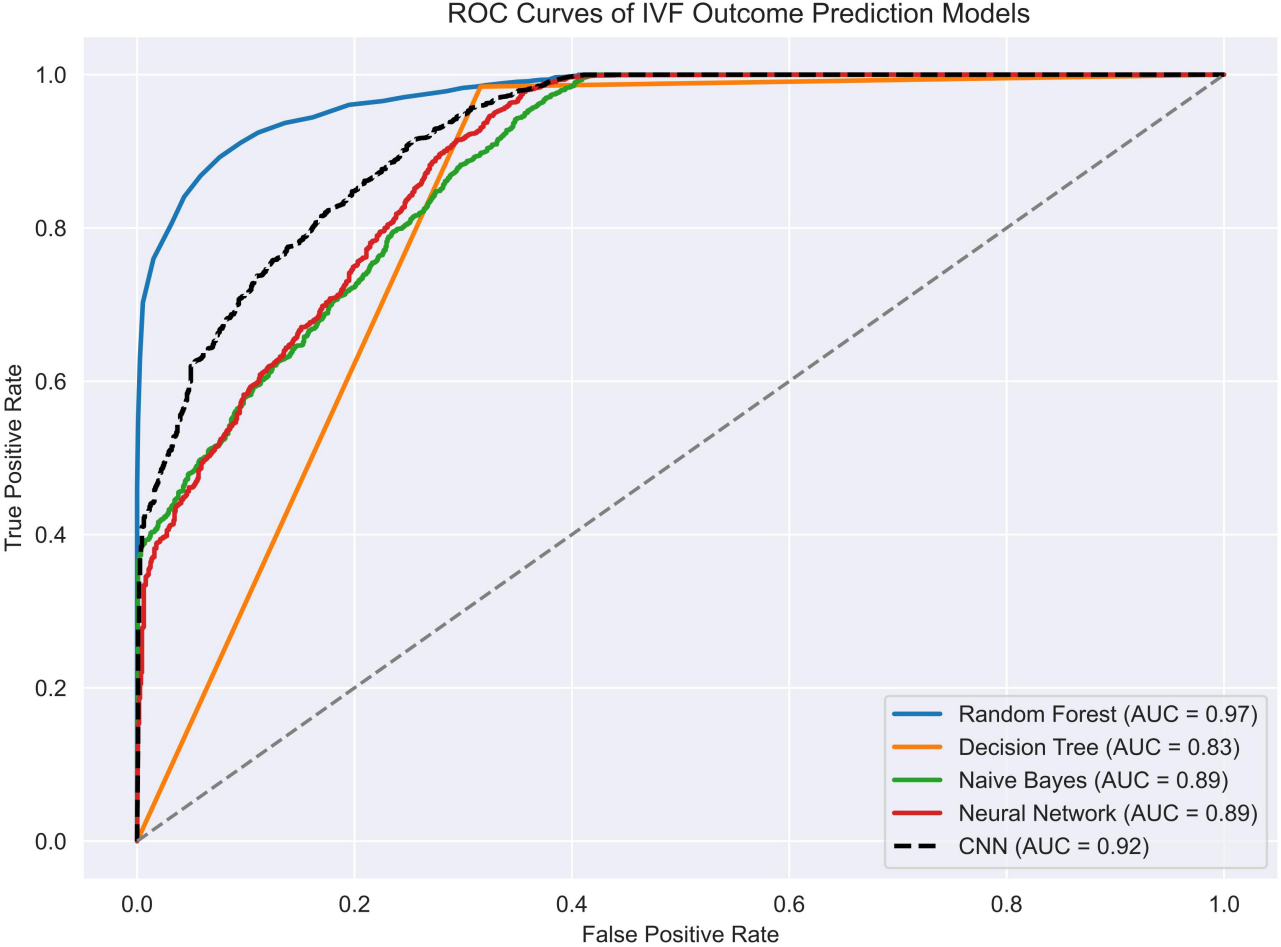
ROC Curves of IVF Outcome Prediction Models.

**Figure 4** compares the receiver operating characteristic (ROC) curves of the five models tested: Random Forest, Decision Tree, Naive Bayes, Feedforward Neural Network, and CNN. Among them, Random Forest demonstrated the highest discriminative power with an AUC of 0.9734, closely followed by CNN and the feedforward neural network.

Notably, CNN achieved excellent recall and sensitivity, while also maintaining high overall accuracy on the testing set. In contrast, Naive Bayes showed significantly poorer classification performance, with a ROC curve approaching the diagonal and accuracy below 50%, indicating limited generalizability.

A summary of quantitative performance metrics for all five models is provided in **Table 1**. Random Forest achieved the highest accuracy (0.9406 ± 0.0017), F1 score (0.9666 ± 0.0009), and AUC (0.9734 ± 0.0012), confirming its robustness in binary classification tasks involving EMR data. The CNN model performed comparably well, with an accuracy of 0.9394 ± 0.0013, F1 score of 0.9660 ± 0.0007, and recall of 0.9993 ± 0.0012, demonstrating its strength in detecting positive outcomes with minimal compromise on precision.

**Table 1:** Experimental results of five models.

Model	Accuracy	AUC	Precision	Recall	F1 Score
Random Forest	0.9406 ± 0.0017	0.9734 ± 0.0012	0.9356 ± 0.0018	0.9997 ± 0.0002	0.9666 ± 0.0009
Decision Tree	0.9387 ± 0.0026	0.8249 ± 0.0051	0.9478 ± 0.0014	0.9829 ± 0.0022	0.9650 ± 0.0015
Naive Bayes	0.4889 ± 0.0138	0.8795 ± 0.0034	0.9892 ± 0.0032	0.4103 ± 0.0173	0.5798 ± 0.0171
Neural Network	0.9315 ± 0.0029	0.8896 ± 0.0041	0.9426 ± 0.0018	0.9801 ± 0.0037	0.9610 ± 0.0017
CNN	0.9394 ± 0.0013	0.8899 ± 0.0032	0.9348 ± 0.0018	0.9993 ± 0.0012	0.9660 ± 0.0007

The feedforward neural network also performed well (accuracy = 0.9315 ± 0.0029), while Naive Bayes underperformed across all metrics, particularly in accuracy (0.4889 ± 0.0138), largely due to its strong assumptions of feature independence and lack of capacity to model nonlinear feature interactions.

## Discussion

### Main findings

In this study, we compared the performance of five machine learning models in predicting live birth outcomes among patients undergoing IVF treatment. Among these, Random Forest and a custom-designed CNN demonstrated superior performance across multiple evaluation metrics, including AUC and F1 score (**Table 1**). The CNN model in particular achieved a near-perfect recall (0.9993 ± 0.0012) and an overall F1 score (0.9660 ± 0.0007), indicating excellent sensitivity in capturing positive outcomes.

**Figure 5:**
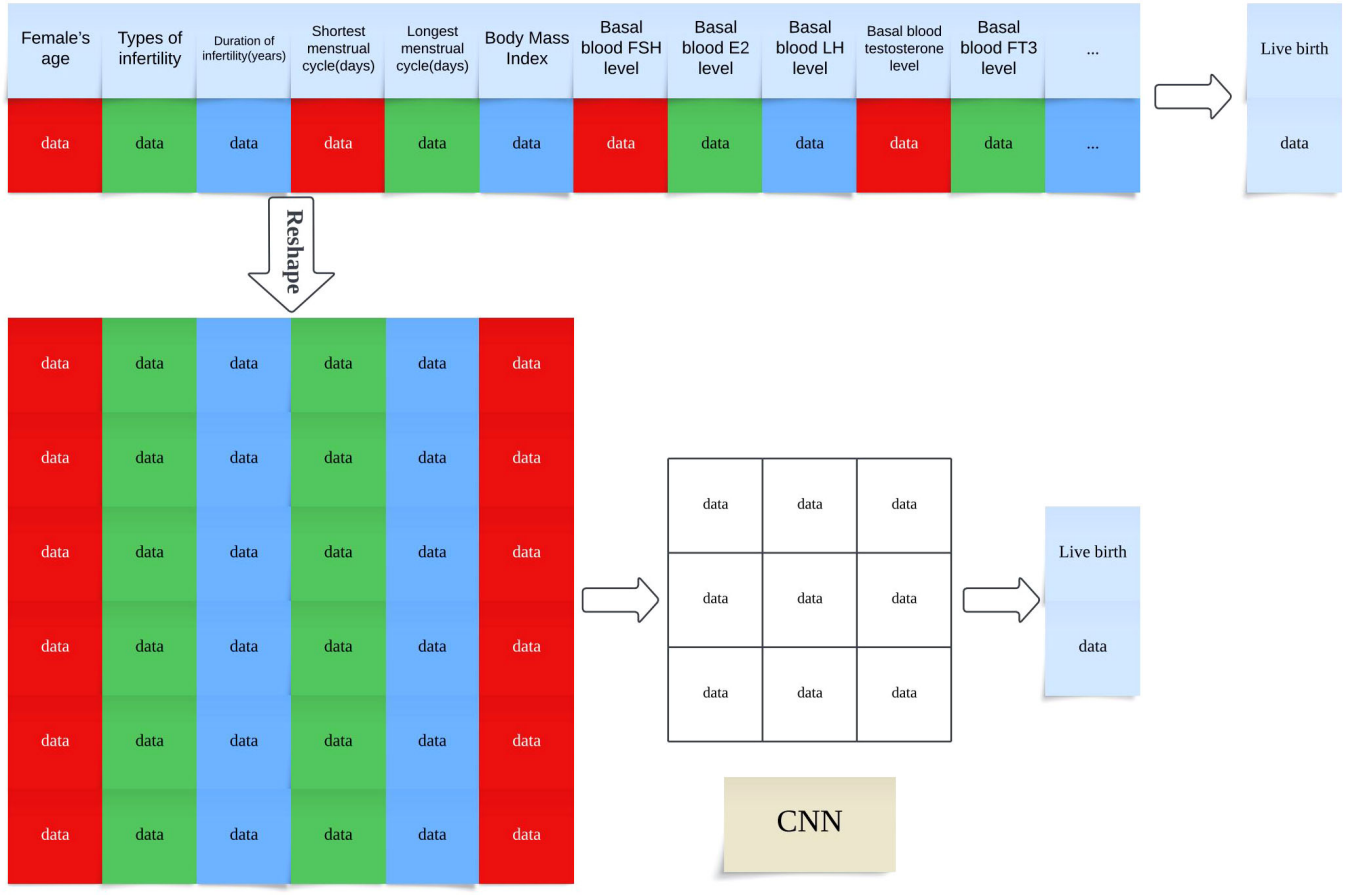
Transformation of Structured EMR Data into CNN-Compatible Matrix Format for Live Birth Prediction Note: Schematic illustration of how structured electronic medical record (EMR) features are reshaped into a two-dimensional matrix to serve as input for convolutional neural network (CNN) training. Each row represents a single patient record, and features are organized spatially to enable convolutional filters to extract local patterns The model predicts the live birth outcome based on the encoded feature matrix.

While CNNs are traditionally applied to image data, their use in this study to model structured EMR data was motivated by several factors (19). First, by reshaping clinical variables into a two-dimensional matrix (**Figure 5**), we enabled the CNN to detect local feature patterns and higher-order interactions among related clinical factors. This spatial modeling paradigm allows the network to simulate implicit relationships—such as those between hormone levels and oocyte quality—that may not be easily captured by traditional machine learning methods with flat input vectors (19, 20).

Second, CNNs offer significant advantages in terms of parameter efficiency and scalability. Compared to fully connected deep networks, convolutional layers require fewer parameters and can generalize well from moderate-sized datasets, such as the one used in this study. Additionally, CNNs are more compatible with future extensions to multimodal data inputs, including ultrasound images, embryo morphokinetics, and time-lapse videos, which are increasingly available in modern IVF practice (19, 20).

Taken together, these findings highlight that with appropriate preprocessing, CNNs can be successfully adapted to structured EMR datasets and achieve robust prediction performance comparable to—or exceeding—traditional machine learning models.

### Strengths and limitations

This study has several strengths. It leverages a large dataset of 48,514 patients, providing a robust foundation for training and evaluating predictive models. The use of both deep learning (CNNs) and traditional machine learning models allows for a comprehensive comparison, highlighting the relative advantages of each approach in analyzing EMR data. Additionally, the integration of XGBoost and SHAP for feature importance analysis enhances the interpretability of the models, offering clinicians valuable insights into the factors influencing live birth outcomes.

However, there are also notable limitations. First, the data was collected from a single medical center, which may limit the generalizability of the findings to other populations and settings. Second, this study did not include multimodal data, such as imaging results, which could potentially improve predictive accuracy but would also require greater computational resources. Millions of people face catastrophic healthcare costs after seeking treatment for infertility, making this a major equity issue and all too often, a medical poverty trap for those affected,” said Pascale Allotey, PhD, MMedSci, the WHO director of sexual and reproductive health and research (21). Socioeconomic factors, such as patients’ financial status and educational background, were not considered, which might influence treatment outcomes and decision-making processes. In economically underdeveloped regions of China, financial constraints pose a significant challenge for many infertile couples, who may also experience heightened anxiety during assisted reproductive treatments. This factor could potentially influence treatment outcomes and clinical decision-making. Since July 2023, healthcare authorities across various regions in China have progressively incorporated assisted reproductive technologies into the national medical insurance system to alleviate the financial burden on patients. Future research exploring how patients’ socioeconomic status and educational background influence ART outcomes—both before and after the implementation of this policy—would be valuable. Addressing this limitation could further validate and expand the applicability of our findings in real-world settings.

### Interpretation

Our findings are consistent with recent research on the use of machine learning (ML) and deep learning (DL) in assisted reproductive technologies. Studies have shown that ML can optimize processes like individualized dosing during ovarian stimulation, which enhances patient outcomes and reduces cost (22). Meanwhile, DL models, particularly those using imaging data, have been successful in predicting embryo viability, offering more accurate and consistent evaluations than traditional approaches (23). These advancements demonstrate how both ML and DL can significantly improve clinical decision-making in ART, making treatments more personalized and efficient. Studies have demonstrated the effectiveness of Random Forests and XGBoost in predicting outcomes for IVF treatments, emphasizing their ability to process and interpret structured clinical data (22). Recent studies have demonstrated that Random Forest and XGBoost models can effectively analyze clinical factors influencing IVF success rates, showing superior performance in handling large-scale, structured datasets (24, 25). Another study highlighted XGBoost’s accuracy in predicting embryo viability and live birth outcomes, attributing its strength to its capacity for managing complex, structured inputs like patient records (26). These studies confirm the value of these models in assisted reproductive technologies. Our results confirm the strong predictive capabilities of these methods while highlighting the potential of CNNs to capture complex relationships within EMR data, a feature that is often less explored in ART research.

Interestingly, while the predictive accuracy of CNNs was slightly lower than that of some traditional models, CNNs provided unique insights into data structure, suggesting that their ability to model spatial relationships could be further harnessed in more complex datasets, such as those incorporating imaging data. This complements studies that have utilized CNNs for embryo assessment, sperm analysis, and other image-based evaluations in fertility clinics.

Our study also sheds light on the feasibility of deploying AI models in resource-limited settings, an area that has received less attention in the literature. The minimal computational demands observed during the analysis of EMR data contrast sharply with the high resource requirements often associated with AI applications in areas like natural language processing (NLP) and medical imaging. For example, large models such as BERT (27) and GPT-3 (28) require substantial GPU resources and energy consumption, making their deployment challenging in settings with limited computational infrastructure. Strubell et al. highlight the significant energy consumption and carbon footprint associated with training deep learning models for NLP, further emphasizing the barriers to deploying such models globally (27). Similarly, Esteva et al. note that AI applications in healthcare, particularly those involving medical imaging, require extensive computational resources, which can limit their use in underdeveloped regions (29). By contrast, our findings demonstrate that AI models designed for EMR analysis can achieve accurate predictions with much lower computational demands, making localized deployment in global reproductive medicine centers more feasible, even in areas with constrained hardware and technical support.

Overall, our study contributes to the growing body of evidence supporting the integration of AI into reproductive medicine. It demonstrates that even data-intensive approaches like CNNs can be effectively adapted for practical application in clinical settings. These findings highlight the importance of future research focused on optimizing different AI architectures for diverse types of clinical data, thereby enhancing predictive performance and ultimately improving patient outcomes.

## Conclusion

This study demonstrates that CNNs can effectively analyze EMRs to predict outcomes in assisted reproductive therapy, achieving performance comparable to traditional models such as Random Forests. Notably, the relatively low computational requirements for training our CNN model suggest that local deployment is feasible even in resource-constrained reproductive centers. These findings underscore the potential of AI to support and enhance clinical decision-making in reproductive medicine. Future studies should aim to validate these results in multicenter settings and investigate the integration of multimodal data to further improve predictive performance and generalizability.

## Data Availability

The data analyzed in this study is subject to the following licenses/restrictions: The data cannot be disclosed because it involves the privacy of the patients. If necessary, the original data can be asked by email, but it cannot be disclosed. Requests to access these datasets should be directed to Yu Liu, zzudoctorliu@163.com.
